# *trans*-Diaqua­bis­(4-fluoro­benzoato-κ*O*)bis­(nicotinamide-κ*N* ^1^)nickel(II)

**DOI:** 10.1107/S1600536811044771

**Published:** 2011-10-29

**Authors:** Hacali Necefoğlu, Vijdan Öztürk, Füreya Elif Özbek, Vedat Adıgüzel, Tuncer Hökelek

**Affiliations:** a Kafkas University, Department of Chemistry, 36100 Kars, Turkey; b Hacettepe University, Department of Physics, 06800 Beytepe, Ankara, Turkey

## Abstract

In the mononuclear Ni^II^ title complex, [Ni(C_7_H_4_FO_2_)_2_(C_6_H_6_N_2_O)_2_(H_2_O)_2_], the Ni^II^ atom, located on an inversion center, is coordinated by two nicotinamide and two 4-fluoro­benzoate ligands and two water mol­ecules in a distorted N_2_O_4_ octa­hedral geometry. The dihedral angle between the carboxyl­ate group and the adjacent benzene ring is 8.95 (8)°, while the pyridine ring and the benzene ring are oriented at a dihedral angle of 75.01 (7)°. The water mol­ecule links the adjacent carboxyl­ate O atom *via* an intra­molecular O—H⋯O hydrogen bond. In the crystal, O—H⋯O, N—H⋯O, C—H⋯O and C—H⋯F hydrogen bonds link the mol­ecules into a three-dimensional network. π–π stacking between parallel pyridine rings [centroid–centroid distance = 3.7287 (11) Å] is also observed.

## Related literature

For literature on niacin, see: Krishnamachari (1974 ▶). For information on the nicotinic acid derivative *N*,*N*-diethyl­nicotinamide, see: Bigoli *et al.* (1972 ▶). For related structures, see: Hökelek *et al.* (1996 ▶, 2009*a* ▶,*b* ▶); Hökelek & Necefoğlu (1998 ▶, 2007) ▶; Necefoğlu *et al.* (2011 ▶). For bond-length data, see: Allen *et al.* (1987 ▶).
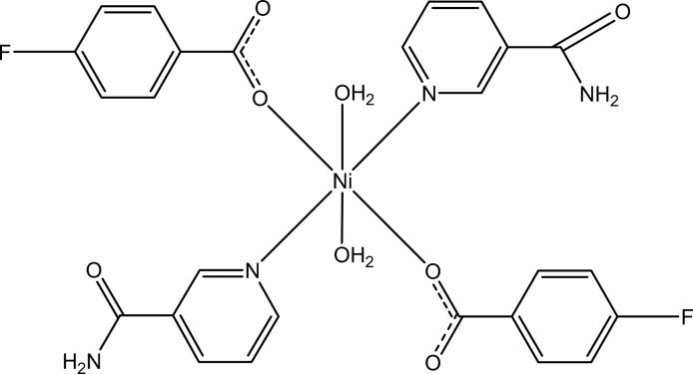


## Experimental

### 

#### Crystal data

[Ni(C_7_H_4_FO_2_)_2_(C_6_H_6_N_2_O)_2_(H_2_O)_2_]
*M* *_r_* = 617.18
Monoclinic, 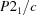

*a* = 12.2001 (5) Å
*b* = 8.8473 (4) Å
*c* = 17.1341 (5) Å
β = 136.080 (2)°
*V* = 1282.86 (10) Å^3^
*Z* = 2
Mo *K*α radiation
μ = 0.83 mm^−1^
*T* = 100 K
0.29 × 0.22 × 0.18 mm

#### Data collection

Bruker Kappa APEXII CCD area-detector diffractometer
Absorption correction: multi-scan (*SADABS*; Bruker, 2005 ▶) *T*_min_ = 0.803, *T*_max_ = 0.861
11926 measured reflections
3220 independent reflections
2874 reflections with *I* > 2σ(*I*)
*R*_int_ = 0.028

#### Refinement

*R*[*F* ^2^ > 2σ(*F* ^2^)] = 0.030
*wR*(*F* ^2^) = 0.077
*S* = 1.04
3220 reflections
203 parameters
H atoms treated by a mixture of independent and constrained refinement
Δρ_max_ = 0.46 e Å^−3^
Δρ_min_ = −0.57 e Å^−3^

### 

Data collection: *APEX2* (Bruker, 2007 ▶); cell refinement: *SAINT* (Bruker, 2007 ▶); data reduction: *SAINT*; program(s) used to solve structure: *SHELXS97* (Sheldrick, 2008 ▶); program(s) used to refine structure: *SHELXL97* (Sheldrick, 2008 ▶); molecular graphics: *ORTEP-3 for Windows* (Farrugia, 1997 ▶); software used to prepare material for publication: *WinGX* (Farrugia, 1999 ▶) and *PLATON* (Spek, 2009 ▶).

## Supplementary Material

Crystal structure: contains datablock(s) I, global. DOI: 10.1107/S1600536811044771/xu5359sup1.cif

Structure factors: contains datablock(s) I. DOI: 10.1107/S1600536811044771/xu5359Isup2.hkl

Additional supplementary materials:  crystallographic information; 3D view; checkCIF report

## Figures and Tables

**Table 1:** Selected bond lengths (Å)

Ni1—O1	2.0500 (9)
Ni1—O4	2.0872 (10)
Ni1—N1	2.1033 (13)

**Table 2:** Hydrogen-bond geometry (Å, °)

*D*—H⋯*A*	*D*—H	H⋯*A*	*D*⋯*A*	*D*—H⋯*A*
N2—H21⋯O3^i^	0.84 (3)	2.15 (3)	2.8363 (19)	139 (2)
N2—H22⋯O4^ii^	0.86 (3)	2.28 (3)	2.955 (2)	135 (2)
O4—H41⋯O3^iii^	0.841 (18)	1.94 (2)	2.7654 (16)	166 (3)
O4—H42⋯O2	0.88 (3)	1.70 (2)	2.5663 (14)	168 (4)
C6—H6⋯O4^iv^	0.93	2.52	3.402 (3)	159
C8—H8⋯F1^v^	0.93	2.53	3.1358 (18)	123
C9—H9⋯F1^v^	0.93	2.55	3.129 (2)	121
C10—H10⋯O2^vi^	0.93	2.57	3.4060 (19)	150

Symmetry codes: (i) 
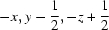
; (ii) 
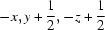
; (iii) 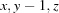; (iv) 
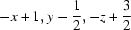
; (v) 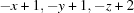; (vi) 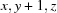.

**Fig. 1.**
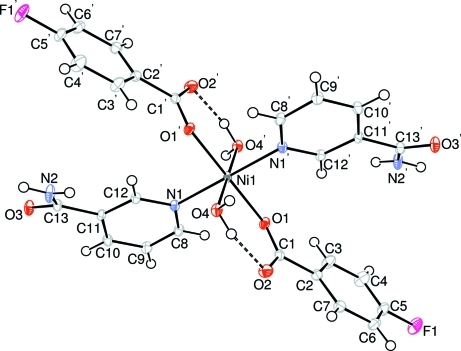
The molecular structure of the title molecule with the atom-numbering scheme. Displacement ellipsoids are drawn at the 50% probability level [symmetry code ('): -x, 1-y, 1-z].

## supplementary crystallographic information

### Comment

As a part of our ongoing investigations of transition metal complexes of nicotinamide (NA), one form of niacin (Krishnamachari, 1974), and/or the nicotinic acid derivative *N*,*N*-diethylnicotinamide (DENA), an important respiratory stimulant (Bigoli *et al.*, 1972), the title compound was synthesized and its crystal structure is reported herein.

The asymmetric unit of the title mononuclear Ni^II^ complex, (Fig. 1), contains one-half molecule, the Ni^II^ atom being located on an inversion center. It consists of two nicotinamide (NA), two 4-fluorobenzoate (PFB) ligands and two coordinated water molecules, all ligands coordinating in a monodentate manner. The crystal structures of similar omplexes of Cu^II^, Co^II^, Ni^II^, Mn^II^ and Zn^II^ ions, [Cu(C_7_H_5_O_2_)_2_(C_10_H_14_N_2_O)_2_] (Hökelek *et al.*, 1996), [Co(C_6_H_6_N_2_O)_2_(C_7_H_4_NO_4_)_2_(H_2_O)_2_] (Hökelek & Necefouglu, 1998), [Co(C_9_H_9_O_2_)_2_(C_10_H_14_N_2_O)_2_(H_2_O)_2_] (Necefoğlu *et al.*, 2011), [Ni(C_7_H_4_ClO_2_)_2_(C_6_H_6_N_2_O)_2_(H_2_O)_2_] (Hökelek *et al.*, 2009*a*), [Mn(C_9_H_10_NO_2_)_2_(H_2_O)_4_].2H_2_O (Hökelek & Necefoğlu, 2007) and [Zn(C_7_H_4_BrO_2_)_2_(C_6_H_6_N_2_O)_2_(H_2_O)_2_] (Hökelek *et al.*, 2009*b*) have also been reported. In the copper(II) complex mentioned above the two benzoate ions coordinate to the Cu^II^ atom as bidentate ligands, while in the other structures all the ligands coordinate in a monodentate manner.

In the title complex,  the four symmetry related O atoms (O1, O1', O4 and O4') in the equatorial plane around the Ni^II^ ion form a slightly distorted square-planar arrangement, while the slightly distorted octahedral coordination is completed by the two N atoms of the NA ligands (N1 and N1') in the axial positions. The near equalities of the C1—O1 [1.2695 (18) Å] and C1—O2 [1.2560 (16) Å] bonds in the carboxylate groups indicate delocalized bonding arrangements, rather than localized single and double bonds. The Ni—O bond lengths are 2.0500 (9) Å (for benzoate oxygen) and 2.0872 (10) Å (for water oxygen), and the Ni—N bond length is 2.1033 (13) Å, close to standard values (Allen *et al.*, 1987). The intramolecular O—H···O hydrogen bonds (Table 1) link the water molecules to the carboxylate groups. The Ni atom is displaced out of the mean-plane of the carboxylate group (O1/C1/O2) by 0.5609 (1) Å. The dihedral angle between the planar carboxylate group and the adjacent benzene ring A (C2—C7) is 8.95 (8)°. The benzene A (C2—C7) and the pyridine B (N1/C8—C12) rings are oriented at a dihedral angle of A/B = 75.01 (7)°.

In the crystal, intermolecular O—H···O, N—H···O, C—H···O and C—H···F hydrogen bonds (Table 1) link the molecules into a three-dimensional network. There also exists a π–π contact between the pyridine rings, Cg2—Cg2^i^, may further stabilize the structure [centroid-centroid distance = 3.729 (1) Å; symmetry code: (i) 2 - x, -y, 1 - z; Cg2 is the centroid of the ring B (N1/C8—C12)].

### Experimental

The title compound was prepared by the reaction of NiSO_4_.6H_2_O (1.31 g, 5 mmol) in H_2_O (25 ml) and NA (1.22 g, 10 mmol) in H_2_O (25 ml) with sodium 4-fluorobenzoate (1.62 g, 10 mmol) in H_2_O (100 ml) at room temperature. The mixture was filtered and set aside to crystallize at ambient temperature for two weeks, giving blue single crystals.

### Refinement

Atoms H41 and H42 (for water molecules) and H21 and H22 (for NH_2_ groups) were located in a difference Fourier map and were freely refined. The C-bound H-atoms were positioned geometrically with C—H = 0.93 Å, for aromatic H-atoms, and constrained to ride on their parent atoms, with *U*_iso_(H) = 1.2*U*_eq_(C).

### Crystal data

**Table:**

[Ni(C_7_H_4_FO_2_)_2_(C_6_H_6_N_2_O)_2_(H_2_O)_2_]	*F*(000) = 636
*M**_r_* = 617.18	*D*_x_ = 1.598 Mg m^−^^3^
Monoclinic, *P*2_1_/*c*	Mo *K*α radiation, λ = 0.71073 Å
Hall symbol: -P 2ybc	Cell parameters from 6642 reflections
*a* = 12.2001 (5) Å	θ = 2.4–28.5°
*b* = 8.8473 (4) Å	µ = 0.83 mm^−^^1^
*c* = 17.1341 (5) Å	*T* = 100 K
β = 136.080 (2)°	Block, blue
*V* = 1282.86 (10) Å^3^	0.29 × 0.22 × 0.18 mm
*Z* = 2	

### Data collection

**Table:**

Bruker Kappa APEXII CCD area-detector diffractometer	3220 independent reflections
Radiation source: fine-focus sealed tube	2874 reflections with *I* > 2σ(*I*)
graphite	*R*_int_ = 0.028
φ and ω scans	θ_max_ = 28.5°, θ_min_ = 2.4°
Absorption correction: multi-scan (*SADABS*; Bruker, 2005)	*h* = −16→16
*T*_min_ = 0.803, *T*_max_ = 0.861	*k* = −11→11
11926 measured reflections	*l* = −22→23

### Refinement

**Table:**

Refinement on *F*^2^	Primary atom site location: structure-invariant direct methods
Least-squares matrix: full	Secondary atom site location: difference Fourier map
*R*[*F*^2^ > 2σ(*F*^2^)] = 0.030	Hydrogen site location: inferred from neighbouring sites
*wR*(*F*^2^) = 0.077	H atoms treated by a mixture of independent and constrained refinement
*S* = 1.04	*w* = 1/[σ^2^(*F*_o_^2^) + (0.038*P*)^2^ + 0.689*P*] where *P* = (*F*_o_^2^ + 2*F*_c_^2^)/3
3220 reflections	(Δ/σ)_max_ < 0.001
203 parameters	Δρ_max_ = 0.46 e Å^−^^3^
0 restraints	Δρ_min_ = −0.57 e Å^−^^3^

### Special details

**Table:**

Geometry. All esds (except the esd in the dihedral angle between two l.s. planes) are estimated using the full covariance matrix. The cell esds are taken into account individually in the estimation of esds in distances, angles and torsion angles; correlations between esds in cell parameters are only used when they are defined by crystal symmetry. An approximate (isotropic) treatment of cell esds is used for estimating esds involving l.s. planes.
Refinement. Refinement of F^2^ against ALL reflections. The weighted R-factor wR and goodness of fit S are based on F^2^, conventional R-factors R are based on F, with F set to zero for negative F^2^. The threshold expression of F^2^ > 2sigma(F^2^) is used only for calculating R-factors(gt) etc. and is not relevant to the choice of reflections for refinement. R-factors based on F^2^ are statistically about twice as large as those based on F, and R- factors based on ALL data will be even larger.

### Fractional atomic coordinates and isotropic or equivalent isotropic displacement parameters (Å^2^)

**Table:**

	*x*	*y*	*z*	*U*_iso_*/*U*_eq_	
Ni1	0.0000	0.5000	0.5000	0.00935 (8)	
O1	0.18950 (11)	0.45525 (13)	0.66805 (8)	0.0129 (2)	
O2	0.35932 (12)	0.35155 (14)	0.66999 (8)	0.0182 (2)	
O3	0.04376 (12)	1.15787 (12)	0.34180 (8)	0.0152 (2)	
O4	0.11757 (12)	0.41708 (13)	0.46215 (8)	0.0127 (2)	
H41	0.088 (2)	0.333 (2)	0.4293 (16)	0.020 (5)*	
H42	0.209 (3)	0.398 (3)	0.532 (2)	0.049 (7)*	
N1	0.08897 (13)	0.71649 (14)	0.52032 (9)	0.0114 (2)	
N2	−0.00057 (17)	0.93579 (17)	0.26006 (11)	0.0180 (3)	
H21	0.003 (2)	0.841 (3)	0.2615 (17)	0.030 (6)*	
H22	−0.033 (3)	0.984 (3)	0.203 (2)	0.037 (6)*	
F1	0.65268 (11)	0.13200 (13)	1.13785 (7)	0.0274 (2)	
C1	0.31188 (15)	0.37925 (17)	0.71362 (11)	0.0124 (3)	
C2	0.40420 (15)	0.31422 (18)	0.82813 (11)	0.0132 (3)	
C3	0.36738 (18)	0.3548 (2)	0.88572 (12)	0.0192 (3)	
H3	0.2866	0.4236	0.8538	0.023*	
C4	0.45092 (19)	0.2929 (2)	0.99065 (12)	0.0229 (4)	
H4	0.4270	0.3190	1.0296	0.027*	
C5	0.56940 (17)	0.1923 (2)	1.03506 (11)	0.0185 (3)	
C6	0.60852 (17)	0.14784 (19)	0.98051 (12)	0.0176 (3)	
H6	0.6885	0.0779	1.0127	0.021*	
C7	0.52424 (16)	0.21111 (18)	0.87575 (11)	0.0152 (3)	
H7	0.5487	0.1840	0.8372	0.018*	
C8	0.18734 (16)	0.78640 (18)	0.62049 (11)	0.0130 (3)	
H8	0.2178	0.7358	0.6813	0.016*	
C9	0.24541 (16)	0.93048 (18)	0.63733 (11)	0.0141 (3)	
H9	0.3144	0.9750	0.7082	0.017*	
C10	0.19927 (17)	1.00725 (17)	0.54715 (12)	0.0130 (3)	
H10	0.2353	1.1048	0.5560	0.016*	
C11	0.09784 (15)	0.93567 (17)	0.44284 (11)	0.0107 (3)	
C12	0.04647 (15)	0.79094 (17)	0.43349 (11)	0.0112 (3)	
H12	−0.0205	0.7430	0.3639	0.013*	
C13	0.04432 (16)	1.01818 (17)	0.34384 (11)	0.0121 (3)	

### Atomic displacement parameters (Å^2^)

**Table:**

	*U*^11^	*U*^22^	*U*^33^	*U*^12^	*U*^13^	*U*^23^
Ni1	0.01259 (13)	0.00808 (15)	0.00725 (12)	−0.00057 (10)	0.00710 (10)	0.00028 (9)
O1	0.0143 (4)	0.0119 (5)	0.0089 (4)	0.0004 (4)	0.0072 (4)	0.0005 (4)
O2	0.0158 (5)	0.0271 (7)	0.0124 (4)	0.0011 (5)	0.0104 (4)	0.0016 (5)
O3	0.0245 (5)	0.0088 (5)	0.0148 (4)	−0.0002 (4)	0.0149 (4)	0.0003 (4)
O4	0.0166 (5)	0.0115 (6)	0.0104 (4)	−0.0011 (4)	0.0098 (4)	−0.0014 (4)
N1	0.0136 (5)	0.0107 (6)	0.0113 (5)	−0.0001 (5)	0.0094 (4)	0.0003 (5)
N2	0.0330 (7)	0.0096 (7)	0.0146 (6)	−0.0006 (6)	0.0183 (6)	0.0000 (5)
F1	0.0278 (5)	0.0348 (6)	0.0115 (4)	0.0072 (5)	0.0114 (4)	0.0094 (4)
C1	0.0127 (6)	0.0111 (7)	0.0097 (5)	−0.0039 (6)	0.0068 (5)	−0.0015 (5)
C2	0.0127 (6)	0.0146 (8)	0.0098 (5)	−0.0017 (6)	0.0073 (5)	−0.0007 (5)
C3	0.0187 (7)	0.0240 (9)	0.0143 (6)	0.0063 (7)	0.0117 (6)	0.0035 (6)
C4	0.0254 (7)	0.0318 (10)	0.0152 (6)	0.0062 (8)	0.0159 (6)	0.0027 (7)
C5	0.0176 (6)	0.0214 (9)	0.0092 (6)	0.0001 (7)	0.0072 (5)	0.0030 (6)
C6	0.0143 (6)	0.0181 (8)	0.0147 (6)	0.0036 (6)	0.0086 (5)	0.0032 (6)
C7	0.0146 (6)	0.0169 (8)	0.0135 (6)	−0.0008 (6)	0.0100 (5)	−0.0004 (6)
C8	0.0154 (6)	0.0130 (8)	0.0103 (5)	0.0009 (6)	0.0091 (5)	0.0007 (5)
C9	0.0164 (6)	0.0140 (8)	0.0103 (5)	−0.0015 (6)	0.0091 (5)	−0.0018 (6)
C10	0.0163 (6)	0.0097 (7)	0.0139 (6)	−0.0016 (6)	0.0112 (6)	−0.0011 (5)
C11	0.0130 (6)	0.0110 (7)	0.0105 (5)	0.0021 (6)	0.0092 (5)	0.0023 (5)
C12	0.0132 (6)	0.0112 (7)	0.0097 (5)	0.0001 (6)	0.0085 (5)	−0.0004 (5)
C13	0.0146 (6)	0.0122 (7)	0.0114 (6)	0.0001 (6)	0.0100 (5)	0.0005 (5)

### Geometric parameters (Å, °)

**Table:**

Ni1—O1	2.0500 (9)	C2—C7	1.386 (2)
Ni1—O1^i^	2.0500 (9)	C3—C4	1.390 (2)
Ni1—O4	2.0872 (10)	C3—H3	0.9300
Ni1—O4^i^	2.0872 (10)	C4—H4	0.9300
Ni1—N1	2.1033 (13)	C5—C4	1.369 (2)
Ni1—N1^i^	2.1033 (13)	C6—C5	1.378 (2)
O1—C1	1.2695 (18)	C6—C7	1.3906 (19)
O2—C1	1.2560 (16)	C6—H6	0.9300
O3—C13	1.2363 (18)	C7—H7	0.9300
O4—H41	0.84 (2)	C8—C9	1.384 (2)
O4—H42	0.88 (3)	C8—H8	0.9300
N1—C8	1.3421 (17)	C9—C10	1.3830 (19)
N1—C12	1.3435 (17)	C9—H9	0.9300
N2—C13	1.3264 (19)	C10—C11	1.3930 (19)
N2—H21	0.84 (2)	C10—H10	0.9300
N2—H22	0.86 (2)	C12—C11	1.383 (2)
F1—C5	1.3570 (16)	C12—H12	0.9300
C1—C2	1.5051 (18)	C13—C11	1.4970 (18)
C2—C3	1.3939 (19)		
			
O1^i^—Ni1—O1	180.0	C4—C3—C2	120.28 (14)
O1—Ni1—O4	92.09 (4)	C4—C3—H3	119.9
O1^i^—Ni1—O4	87.91 (4)	C3—C4—H4	120.9
O1—Ni1—O4^i^	87.91 (4)	C5—C4—C3	118.16 (13)
O1^i^—Ni1—O4^i^	92.09 (4)	C5—C4—H4	120.9
O1—Ni1—N1	91.03 (4)	F1—C5—C4	118.72 (13)
O1^i^—Ni1—N1	88.97 (4)	F1—C5—C6	117.82 (14)
O1—Ni1—N1^i^	88.97 (4)	C4—C5—C6	123.46 (13)
O1^i^—Ni1—N1^i^	91.03 (4)	C5—C6—C7	117.73 (14)
O4—Ni1—O4^i^	180.00 (5)	C5—C6—H6	121.1
O4—Ni1—N1	89.05 (4)	C7—C6—H6	121.1
O4^i^—Ni1—N1	90.95 (4)	C2—C7—C6	120.67 (13)
O4—Ni1—N1^i^	90.95 (4)	C2—C7—H7	119.7
O4^i^—Ni1—N1^i^	89.05 (4)	C6—C7—H7	119.7
N1^i^—Ni1—N1	180.00 (8)	N1—C8—C9	122.88 (13)
C1—O1—Ni1	127.09 (9)	N1—C8—H8	118.6
Ni1—O4—H41	117.0 (13)	C9—C8—H8	118.6
Ni1—O4—H42	97.2 (15)	C8—C9—H9	120.5
H41—O4—H42	105 (2)	C10—C9—C8	118.96 (13)
C8—N1—Ni1	120.72 (9)	C10—C9—H9	120.5
C8—N1—C12	117.81 (13)	C9—C10—C11	118.73 (14)
C12—N1—Ni1	121.44 (9)	C9—C10—H10	120.6
C13—N2—H21	123.2 (14)	C11—C10—H10	120.6
C13—N2—H22	116.9 (15)	C10—C11—C13	119.43 (13)
H21—N2—H22	120 (2)	C12—C11—C10	118.63 (12)
O1—C1—C2	116.65 (12)	C12—C11—C13	121.92 (12)
O2—C1—O1	125.40 (12)	N1—C12—C11	122.98 (12)
O2—C1—C2	117.93 (13)	N1—C12—H12	118.5
C3—C2—C1	120.28 (13)	C11—C12—H12	118.5
C7—C2—C1	120.01 (12)	O3—C13—N2	121.96 (13)
C7—C2—C3	119.70 (13)	O3—C13—C11	120.56 (12)
C2—C3—H3	119.9	N2—C13—C11	117.48 (14)
			
O4—Ni1—O1—C1	−10.42 (12)	O2—C1—C2—C7	−8.4 (2)
O4^i^—Ni1—O1—C1	169.58 (12)	C1—C2—C3—C4	178.94 (15)
N1—Ni1—O1—C1	−99.50 (12)	C7—C2—C3—C4	0.3 (2)
N1^i^—Ni1—O1—C1	80.50 (12)	C1—C2—C7—C6	−178.74 (14)
O1—Ni1—N1—C8	−24.63 (11)	C3—C2—C7—C6	−0.1 (2)
O1^i^—Ni1—N1—C8	155.37 (11)	C2—C3—C4—C5	0.2 (3)
O1—Ni1—N1—C12	157.13 (10)	F1—C5—C4—C3	179.47 (15)
O1^i^—Ni1—N1—C12	−22.87 (10)	C6—C5—C4—C3	−1.0 (3)
O4—Ni1—N1—C8	−116.70 (11)	C7—C6—C5—F1	−179.28 (14)
O4^i^—Ni1—N1—C8	63.30 (11)	C7—C6—C5—C4	1.2 (3)
O4—Ni1—N1—C12	65.06 (10)	C5—C6—C7—C2	−0.6 (2)
O4^i^—Ni1—N1—C12	−114.94 (10)	N1—C8—C9—C10	0.7 (2)
Ni1—O1—C1—O2	20.1 (2)	C8—C9—C10—C11	−1.0 (2)
Ni1—O1—C1—C2	−158.24 (10)	C9—C10—C11—C12	0.4 (2)
Ni1—N1—C8—C9	−178.10 (10)	C9—C10—C11—C13	178.87 (13)
C12—N1—C8—C9	0.2 (2)	N1—C12—C11—C10	0.5 (2)
Ni1—N1—C12—C11	177.43 (10)	N1—C12—C11—C13	−177.86 (12)
C8—N1—C12—C11	−0.86 (19)	O3—C13—C11—C10	−23.7 (2)
O1—C1—C2—C3	−8.6 (2)	O3—C13—C11—C12	154.64 (13)
O1—C1—C2—C7	170.00 (14)	N2—C13—C11—C10	155.73 (14)
O2—C1—C2—C3	172.92 (14)	N2—C13—C11—C12	−25.9 (2)

Symmetry codes: (i) −*x*, −*y*+1, −*z*+1.

### Hydrogen-bond geometry (Å, °)

**Table:**

*D*—H···*A*	*D*—H	H···*A*	*D*···*A*	*D*—H···*A*
N2—H21···O3^ii^	0.84 (3)	2.15 (3)	2.8363 (19)	139 (2)
N2—H22···O4^iii^	0.86 (3)	2.28 (3)	2.955 (2)	135 (2)
O4—H41···O3^iv^	0.841 (18)	1.94 (2)	2.7654 (16)	166 (3)
O4—H42···O2	0.88 (3)	1.70 (2)	2.5663 (14)	168 (4)
C6—H6···O4^v^	0.93	2.52	3.402 (3)	159
C8—H8···F1^vi^	0.93	2.53	3.1358 (18)	123
C9—H9···F1^vi^	0.93	2.55	3.129 (2)	121
C10—H10···O2^vii^	0.93	2.57	3.4060 (19)	150

Symmetry codes: (ii) −*x*, *y*−1/2, −*z*+1/2; (iii) −*x*, *y*+1/2, −*z*+1/2; (iv) *x*, *y*−1, *z*; (v) −*x*+1, *y*−1/2, −*z*+3/2; (vi) −*x*+1, −*y*+1, −*z*+2; (vii) *x*, *y*+1, *z*.
